# High mobility group box 1 promotes radioresistance in esophageal squamous cell carcinoma cell lines by modulating autophagy

**DOI:** 10.1038/s41419-019-1355-1

**Published:** 2019-02-12

**Authors:** Hongbing Ma, Shuyu Zheng, Xiaozhi Zhang, Tuotuo Gong, Xin Lv, Shenbo Fu, Shuqun Zhang, Xiaoran Yin, Jingcan Hao, Changyou Shan, Shan Huang

**Affiliations:** 1 Department of Radiation Oncology, Second Affiliated Hospital, Xi’an Jiaotong University, 710004 Xi’an, China; 2 Institute for Molecular Medicine Finland (FIMM), Helsinki Institute of Life Science, University of Helsinki, FI-00014 Helsinki, Finland; 3 Department of Radiation Oncology, First Affiliated Hospital, Xi’an Jiaotong University, 710061 Xi’an, China; 4 Department of Respiratory and Clinical Care Medicine, Second Affiliated Hospital, Xi’an Jiaotong University, 710004 Xi’an, China; 5 Department of Oncology, Second Affiliated Hospital, Xi’an Jiaotong University, 710004 Xi’an, China; 6 Department of Cancer Center, First Affiliated Hospital, Xi’an Jiaotong University, 710061 Xi’an, China

## Abstract

Resistance to radiotherapy results in relapse and treatment failure in locally advanced esophageal squamous cell carcinoma (ESCC). High mobility group box 1 (HMGB1) is reported to be associated with the radioresistance in bladder and breast cancer. However, the role of HMGB1 in the radiotherapy response in ESCC has not been fully elucidated. Here, we investigated the role of HMGB1 to radioresistance in ESCC clinical samples and cell lines. We found that HMGB1 expression was associated with tumor recurrence after postoperative radiotherapy in locally advanced ESCC patients. HMGB1 knockdown in ESCC cells resulted in increased radiosensitivity both in vitro and in vivo. Autophagy level was found depressed in HMGB1 inhibition cells and activation of autophagy brought back cell’s radioresistance. Our results demonstrate that HMGB1 activate autophagy and consequently promote radioresistance. HMGB1 may be used as a predictor of poor response to radiotherapy in ESCC patients. Our finding also highlights the importance of the utility of HMGB1 in ESCC radiosensitization.

## Introduction

Esophageal cancer is the ninth most common malignancy and ranks sixth in cancer deaths worldwide in 2013^1^. Esophageal squamous cell carcinoma (ESCC) is the major histological subtype of esophageal cancer in China^2^. The 5-year overall survival rate of ESCC is 15–25%. For the patients diagnosed at the locally advanced stage, the prognosis is even worse^3^. Preoperative chemoradiotherapy followed by esophagectomy has become the preferred approach for locally advanced esophageal cancer based according to the NCCN guidelines. However, for patients with ESCC undergoing upfront esophagectomy, the optimal postoperative treatment protocol is controversial. Several randomized trials showed no survival benefit for ESCC patients receiving postoperative radiotherapy (PORT)^4,5^. Two large trials by Chen^6^ and Xiao^7^, on the other hand, found that PORT significantly improved the survival of patients with stage III, node-positive ESCC. A certain subgroup of ESCC patients may be more resistant to radiotherapy and obtain little benefit from PORT. However, this group could not be well characterized based on the current clinical and pathological criteria. Investigating the related biomarker has the potential to help the clinicians to tailor the treatment plan for individual ESCC patients. Studying the underlying mechanism may also help to develop effective drug to increase radiosensitivity in these patients.

High mobility group box 1 (HMGB1) is a major family member of injury-related molecules (DAMPs) involving in infection, injury and inflammation^8^. Recently, HMGB1 was reported to be associated with the radioresistance in bladder cancer^9^ and breast cancer^10^. It influences the tumors’ response of radiotherapy possibly through the regulating of DNA damage repair pathways, apoptosis and intracellular autophagy. In ESCC patients, studies have found that the prognosis is negatively correlated with HMGB1 expression in tumor tissues and serum samples^11,12^. However, the role of HMGB1 in the radiotherapy response in ESCC has not been fully elucidated.

In this work, we showed that high HMGB1 expression in tumor tissue is associated with recurrence after PORT for locally advanced resected ESCC. We further investigated the function and the mechanism of HMGB1 in radiotherapy by showing that HMGB1 inhibition increased the radiosensitivity of ESCC both in vitro and in vivo. Mechanistically, HMGB1 inhibition induces low autophagy level, which may contribute to such radiosensitization.

## Results

### HMGB1 expression associates with recurrence after postoperative radiotherapy in locally advanced resected ESCC

We collected in total 120 patients (111 male and 9 female) with locally advanced ESCC. Clinicopathological factors for the 111 male recruited patients were listed in Supplementary Table S1. Among the 111 patients, 42 had in-field recurrence after PORT (37.84%). We examined the association of tumor HMGB1 expression with in-field recurrence after PORT which may reflect tumor radioresistance.

**Fig. 1:**
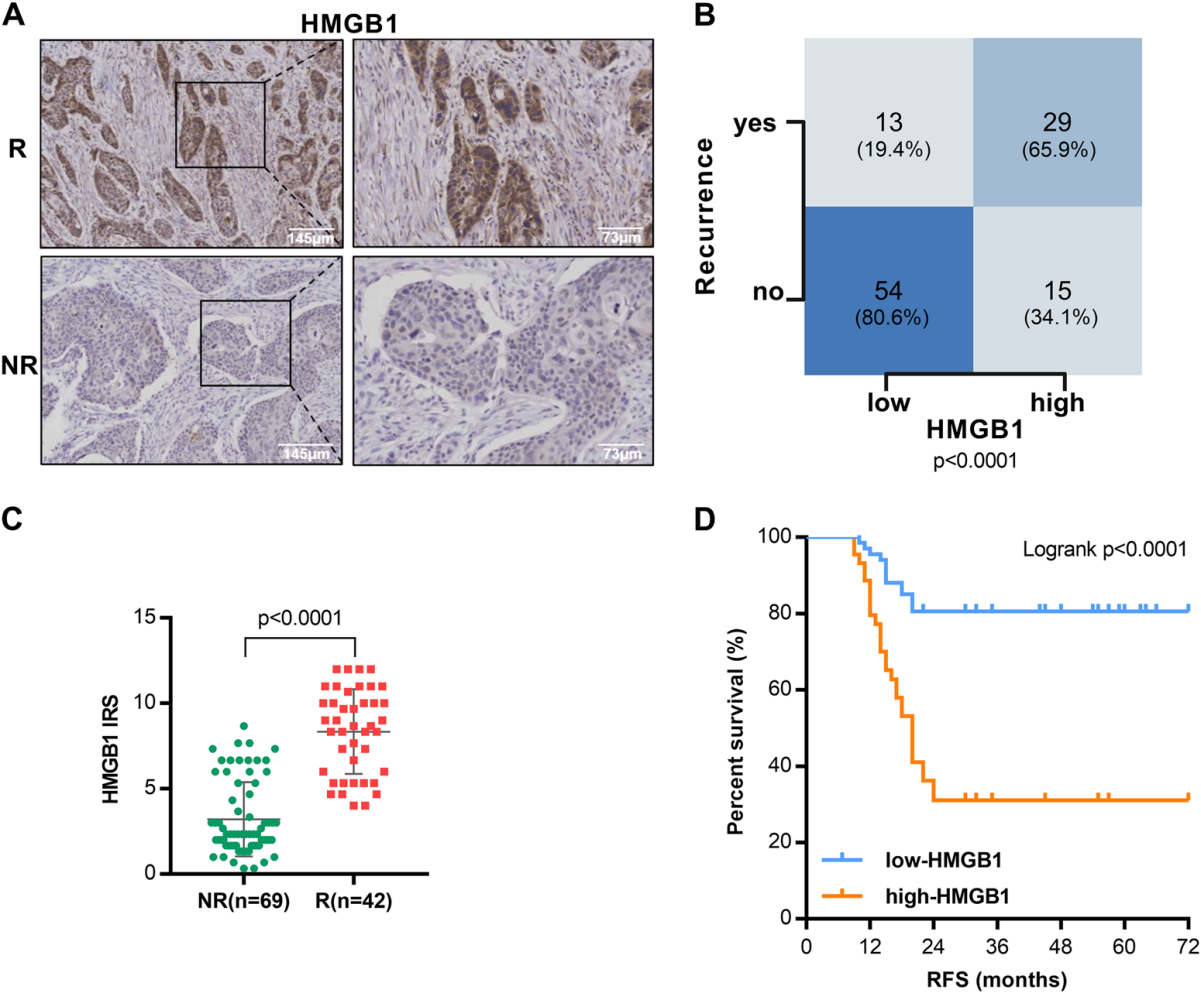
High HMGB1 expression associates with recurrence and poor outcome after postoperative radiotherapy in locally advanced resected ESCC. **a** Representative images of immunohistochemical (IHC) staining of HMGB1 protein in clinical ESCC samples. R, recurrence; NR, non-recurrence. **b** Tumor expression of HMGB1 and recurrence rate in clinical ESCC samples, Chi-square test. **c** Immunoreactivity score (IRS) of HMGB1 protein in clinical ESCC samples, unpaired Mann–Whitney *U* test. **d** Kaplan-Meier analyses of RFS for ESCC with high- or low-level tumor expression of HMGB1, Log-rank test

HMGB1 expression in ESCC tissues was measured by immunohistochemical (IHC) staining (Fig. 1a). Among the male patients, high HMGB1 expression trended towards higher in-field recurrence rate (*P* < 0.0001) (Fig. 1b). The level of tumor HMGB1 expression in recurrence male patients was increased (*P* < 0.0001) (Fig. 1a, c and Supplementary Fig. S1). The preliminary result in female patients was consistent with the male patients (Supplementary Fig. S2). Further Kaplan-Meier analyses showed that high HMGB1 associated with shorter relapse-free survival (RFS) (*P* < 0.0001, Log-rank test, Fig. 1d), consistent with previous published result^11^. Moreover, multivariate analyses revealed that HMGB1 high-expression were independent, unfavorable prognostic indicators for RFS (HR = 3.832, *P* < 0.001) (Supplementary Table S2). At the same time, we have conducted the analysis by considering HMGB1 status as a continues variable (immunoreactivity score, IRS). As presented by Supplementary Table S3, comparison of regression results before and after adjustment confirmed the robust association between tumor HMGB1 expression and survival. These results suggest that HMGB1 expression is clinically relevant to the in-field recurrence of locally advanced resected ESCC.

### HMGB1 knockdown sensitizes ESCC cells to irradiation in vitro and in vivo

**Fig. 2:**
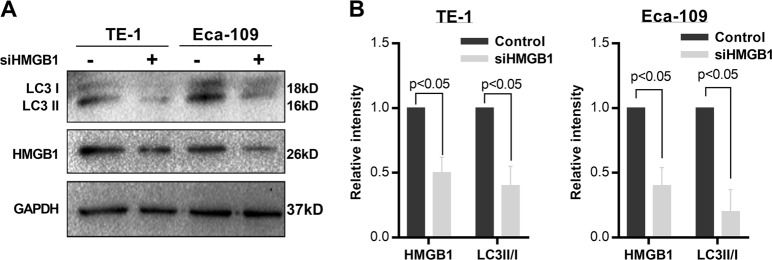
HMGB1 knockdown inhibits the autophagy protein expression in ESCC cells. **a** Western blot was performed to detect LC3 I, LC3 II, and HMGB1 protein expression in siHMGB1- transfected cells. **b** Relative protein density according to GAPDH was analyzed by NIH-Image J, *t* test

**Fig. 3:**
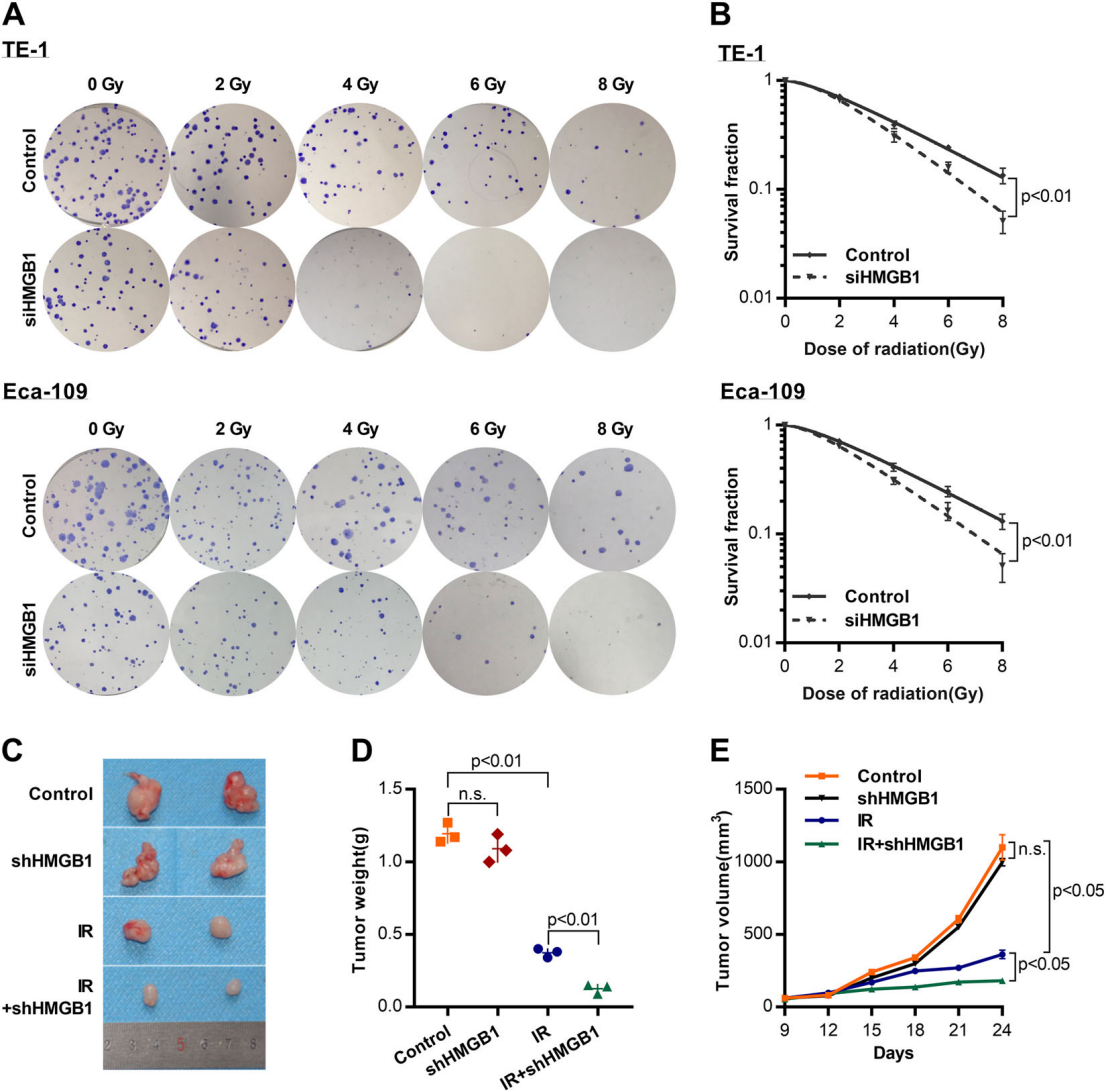
HMGB1 knockdown sensitizes ESCC cells to irradiation in vitro and in vivo. **a** Clonogenic survival assays were performed to measure the radiosensitivity using GraphPad Prism 7.0. **b** Survival curves of TE-1 or Eca-109 treated with irradiation (IR), *t*-test. **c** Representative data of tumors in nude mice bearing TE-1 cells. **d** Tumor weight of xenograft mouse tumors, *t* test. **e** Tumor volumes of xenograft mouse tumors, *t* test

Based on the result that HMGB1 upregulation was association with recurrence after radiotherapy, we hypothesized that HMGB1 knockdown would sensitize ESCC cells to irradiation (IR). To test this, we knocked down HMGB1 expression in two ESCC cell lines (TE-1 and Eca-109) with siRNA oligos (siHMGB1) targeting the HMGB1 gene. Cells were then irradiated by X-rays before seeding on cell culture plates for clonogenic survival assays. The knock down efficiency of three HMGB1 siRNAs was tested by real-time polymerase chain reaction (PCR). We observed highest efficiency for the second siRNA (Supplementary Fig. S3) and used it in the subsequent analysis. Western blot analysis showed that HMGB1 was successfully depleted by siRNA (Fig. 2). Clonogenic survival assays showed that HMGB1 knockdown ESCC cells were more sensitive to IR than control (*P* < 0.05) (Fig. 3a, b). Detailed radiobiological parameters were shown in Table 1, which confirmed the results.

**Table 1:** Radiation biologic parameters of ESCC cells in different groups

Cell lines	Group	*D*_*0*_ (Gy)	*D*_*q*_ (Gy)	*N*	SF2 (%)
TE-1	Control	3.34 ± 0.24	1.88 ± 0.17	1.76 ± 0.41	72.01 ± 2.50
	siHMGB1	2.47 ± 0.31^a,^*	1.83 ± 0.11^a,^*	2.10 ± 0.38^a,^ *	69.08 ± 3.70 ^a,^*
	siHMGB1 + EBSS	3.55 ± 0.26^b,*^	2.58 ± 0.21^b,^*	2.07 ± 0.35^b,^*	80.11 ± 1.70^b,^*
	EBSS	3.64 ± 0.27	2.81 ± 0.18	2.16 ± 0.37	83.10 ± 1.24
Eca-109	Control	3.18 ± 0.19	1.47 ± 0.31	1.59 ± 0.04	70.32 ± 3.30
	siHMGB1	2.48 ± 0.32^a,^*	1.44 ± 0.19^a,^*	1.79 ± 0.22^a,^ *	65.71 ± 4.50^a,^*
	siHMGB1 + EBSS	3.88 ± 0.41^b,^*	2.00 ± 0.23^b,^ *	1.66 ± 0.33^b,^ *	75.11 ± 2.90^b,^*
	EBSS	3.98 ± 0.26	2.38 ± 0.21	1.82 ± 0.39	78.14 ± 1.40

*Note*: Radiobiological parameters (*D*_*0*_, *D*_*q*_, *N* and SF2) were calculated from the single-hit multitarget model (SF = 1−(1−e^ [−kD]) ^N) using GraphPad Prism 7.0

*D*_*0*_: final slope; *D*_*q*_: quasi-threshold dose; *N*: extrapolation number; SF2: survival fraction of 2 Gy

^*^P < 0.05, *t* test

^a^siHMGB1 vs. control

^b^siHMGB1 + EBSS vs. siHMGB1

To validate whether the influence was applicable in vivo, we constructed xenograft mouse model using stably knockdown ESCC cell lines. HMGB1 was stably knocked down in TE-1 cells via retroviral shRNA constructs. TE-1 cells transfected with shHMGB1 or control shRNA were used to establish the ESCC subcutaneous Xenograft mouse model and then the tumor bearing mice were treated with IR. We observed the significantly inhibited tumor growth by IR treatment. In comparison, tumors grew even slower with both IR treatment and HMGB1 depletion in term of tumor weight (*P* < 0.01) and tumor volume (*P* < 0.01) (Fig. 3c–e), indicating the additional suppressive effect brought by HMGB1 depletion. Together with in *vitro* experiments, these results proved that HMGB1 knockdown sensitized ESCC cells lines to IR and that HMGB1 inhibitor might be developed as drug to increase radiotherapy effect in ESCC patients.

### HMGB1 knockdown suppresses the level of autophagy in ESCC cells

Previous studied reported that endogenous HMGB1 can regulate autophagy in human pancreatic tumor cell^13^, which can protect cells from IR-induced damage^14,15^. Our hypothesis was that HMGB1 depletion in ESCC may increase the ESCC’s radiosensitivity through regulating autophagy. This hypothesis can be tested into two steps, first by investigating whether HMGB1 regulates autophagy in ESCC and second, whether such regulation is the key mechanism of elevated radiosensitivity brought by HMGB1 inhibition.

**Fig. 4:**
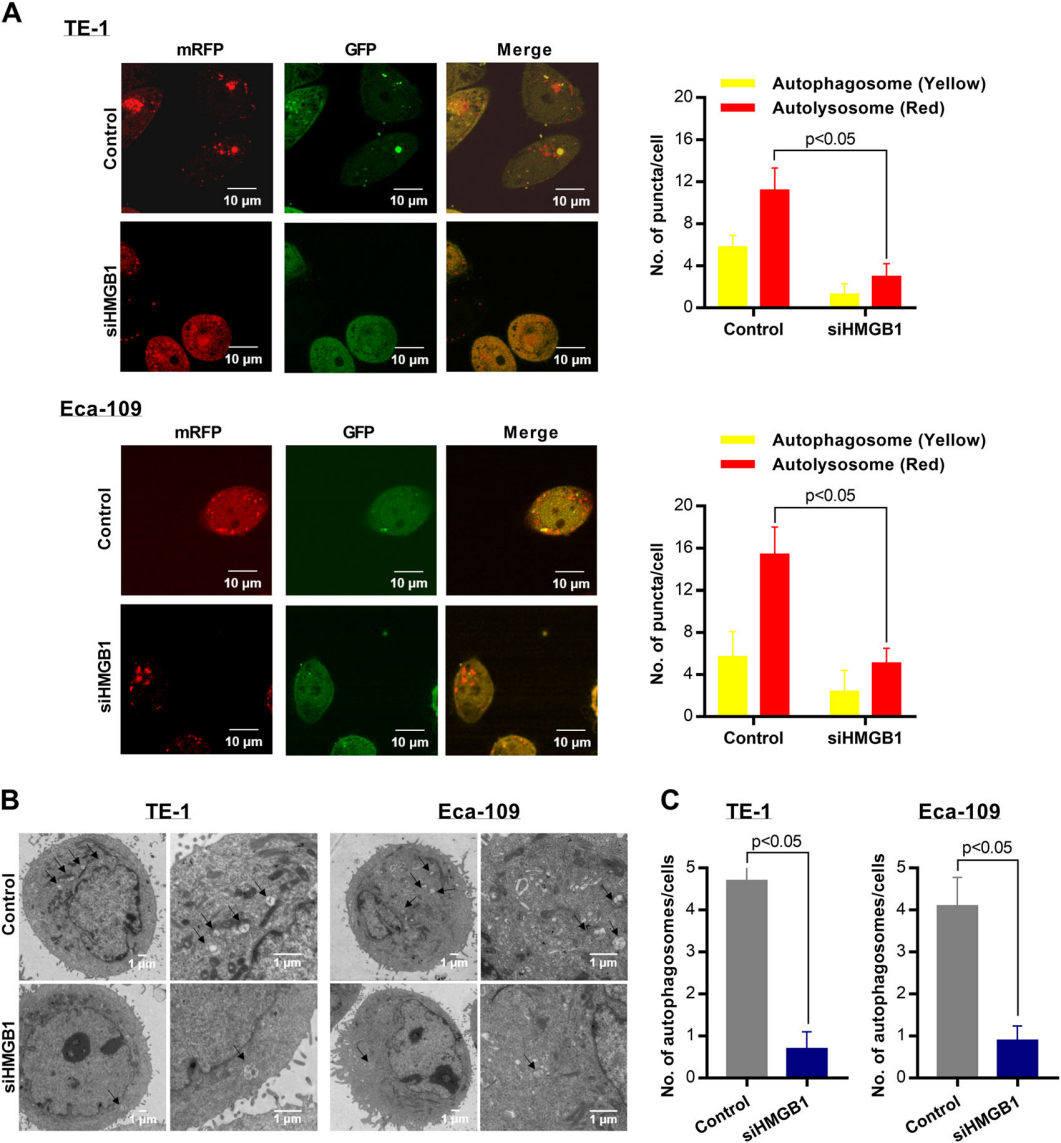
HMGB1 knockdown suppresses the level of autophagy in ESCC cells. **a** ESCC cells stably transfected with mRFP-GFP-LC3 and treated with siHMGB1. Laser confocal fluorescence microscopy analyses (left) and puncta-based quantification (right) for autophagosomes and autolysosomes (*t* test). In the merged image, yellow puncta indicate autophagosomes, while red puncta indicate autolysosomes. **b** TEM microscopic images of autophagosomes in TE-1 and Eca-109 transfected with siHMGB1. **c** Average number of autophagosomes per cell in TE-1 and ECA-109 cells (*t* test)

We tested whether HMGB1 regulate autophagy in ESCC by investigating the level of LC3 II/I in HMGB1 inhibition ESCC cells. LC3 I and LC3 II are established indicators for autophagy and LC3 I will be transformed into LC3 II during autophagy activation^16^. We observed decreased LC3 II and elevated LC3 I in siHMGB1-transfected ESCC cells comparing with control cells, indicating the decreased autophagy level in HMGB1 inhibition ESCC (*P* < 0.05) (Fig. 2). Further fluorescence assay using tandem fluorescent-tagged LC3 (mRFP-GFP-LC3) also observed reduced autophagy flux after siHMGB1 transfection in both TE-1 cells and Eca-109 cells (Fig. 4a). We also employed transmission electron microscopy (TEM), which served as another convincible way to classify the autophagosomes inside cells^16^. TEM images showed that HMGB1 depleted ESCC cells exhibited fewer autophagosomes when compared with controls cells (*P* < 0.05) (Fig. 4b, c). Together our results showed that HMGB1 knockdown reduced the level of autophagy in ESCC cells.

### Activation of autophagy reverses HMGB1-knockdown induced radiosensitization

**Fig. 5:**
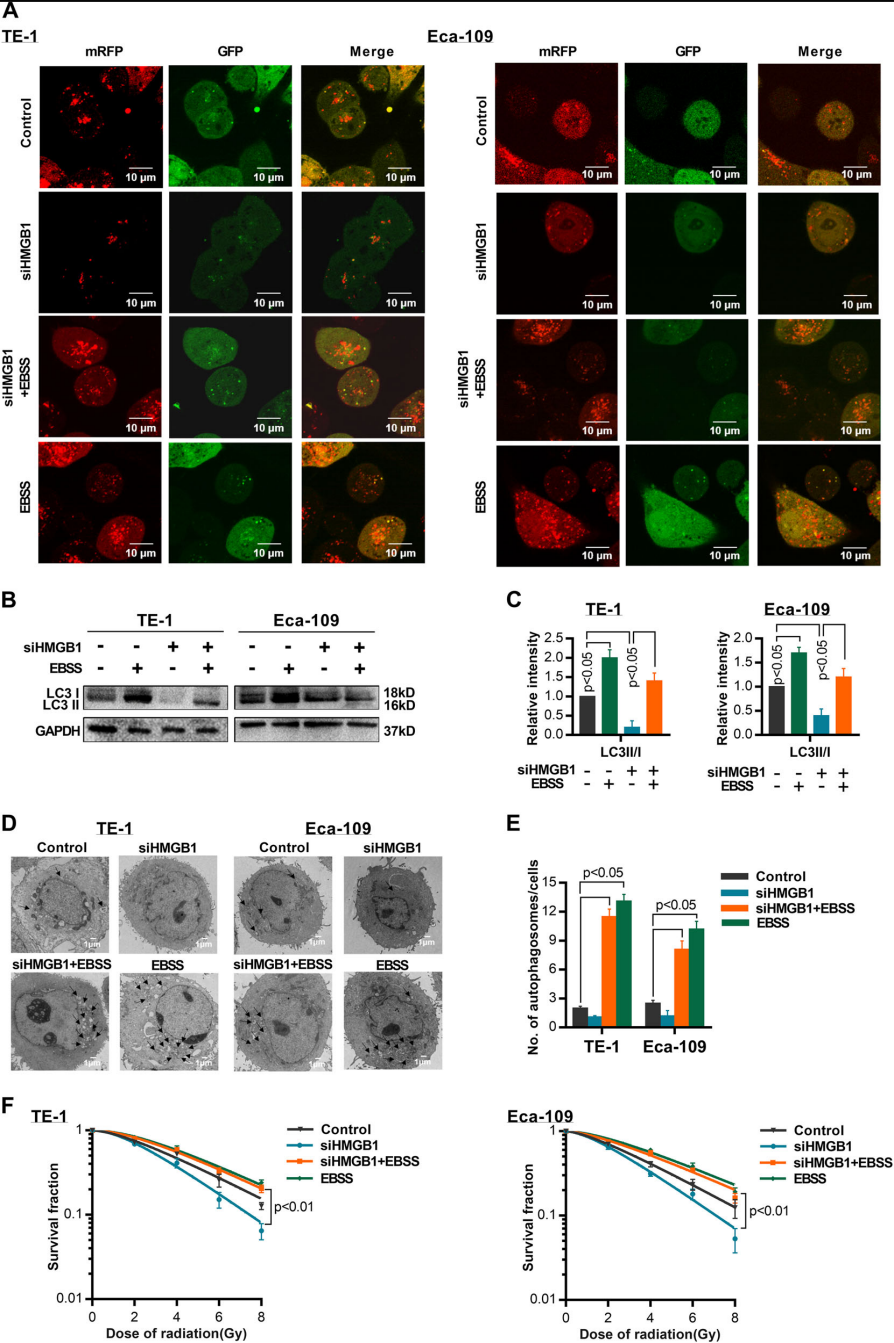
Activation of autophagy reverses radiosensitization induced by HMGB1-knockdown. **a** ESCC cells stably expressed mRFP-GFP-LC3 protein were transfected with siHMGB1 or treated with starvation (EBSS for 4 h). Laser confocal fluorescence microscopy analyses for autophagosomes and autophagosomes. In the merged image, yellow puncta indicate autophagosomes, while red puncta indicate autolysosomes. **b** Western blot was performed to detect LC3 I, LC3 II, and HMGB1 protein expression in siHMGB1-transfected TE-1 or Eca-109 cells and treated with starvation (EBSS for 4 h). **c** Relative protein density according to GAPDH was analyzed by NIH-Image J, *t* test. **d** TEM was used to observe the autophagosomes in siHMGB1-transfected TE-1 or Eca-109 cells and treated with starvation (EBSS for 4 h). **e** The number of autophagosomes in TE-1 and ECA-109 cells under different conditions. **f** Survival curves of TE-1 and Eca-109 treated with IR (0, 2,4,6,8 Gy)

Having proved that HMGB1’s regulation of autophagy in ESCC cells, we then test whether such regulation is the key mechanism for HMGB1’s impact on radiosensitivity. We used starvation (Earle’s Balanced Salt Solution, EBSS) to revert the level of autophagy in siHMGB1-transfected ESCC cells. We observed elevated autophagy flux (Fig. 5a and Supplementray Fig. S4), increased LC3 II and decreased LC3 I expression (*P* < 0.05) (Fig. 5b, c), as well as accumulation of autophagosomes (*P* < 0.05) (Fig. 5d, e) in siHMGB1–transfected ESCC cells after EBSS treatment, indicating that EBSS successfully recovered the from autophagy activation.

After confirmation of autophagy recovery, cells were exposed to various dose of IR treatment. As shown in Fig. 5f, after EBSS treatment, both cell lines presented elevated survival rate, indicating the radioresistance brought by autophagy recovery. Detailed radiobiological parameters of the cells were shown in Table 1, which confirmed the results. Intriguingly, the survival rate of HMGB1 knockdown cells was even higher than controls after EBSS treatment, suggesting that autophagy has a strong regulation effect on radiosensitivity of ESCC. Taken together, our results indicate that siHMGB1 induced radiosensitization is at least partially due to decreased level of autophagy.

### Autophagy level is correlated with expression of HMGB1 and prognosis of ESCC patients

**Fig. 6:**
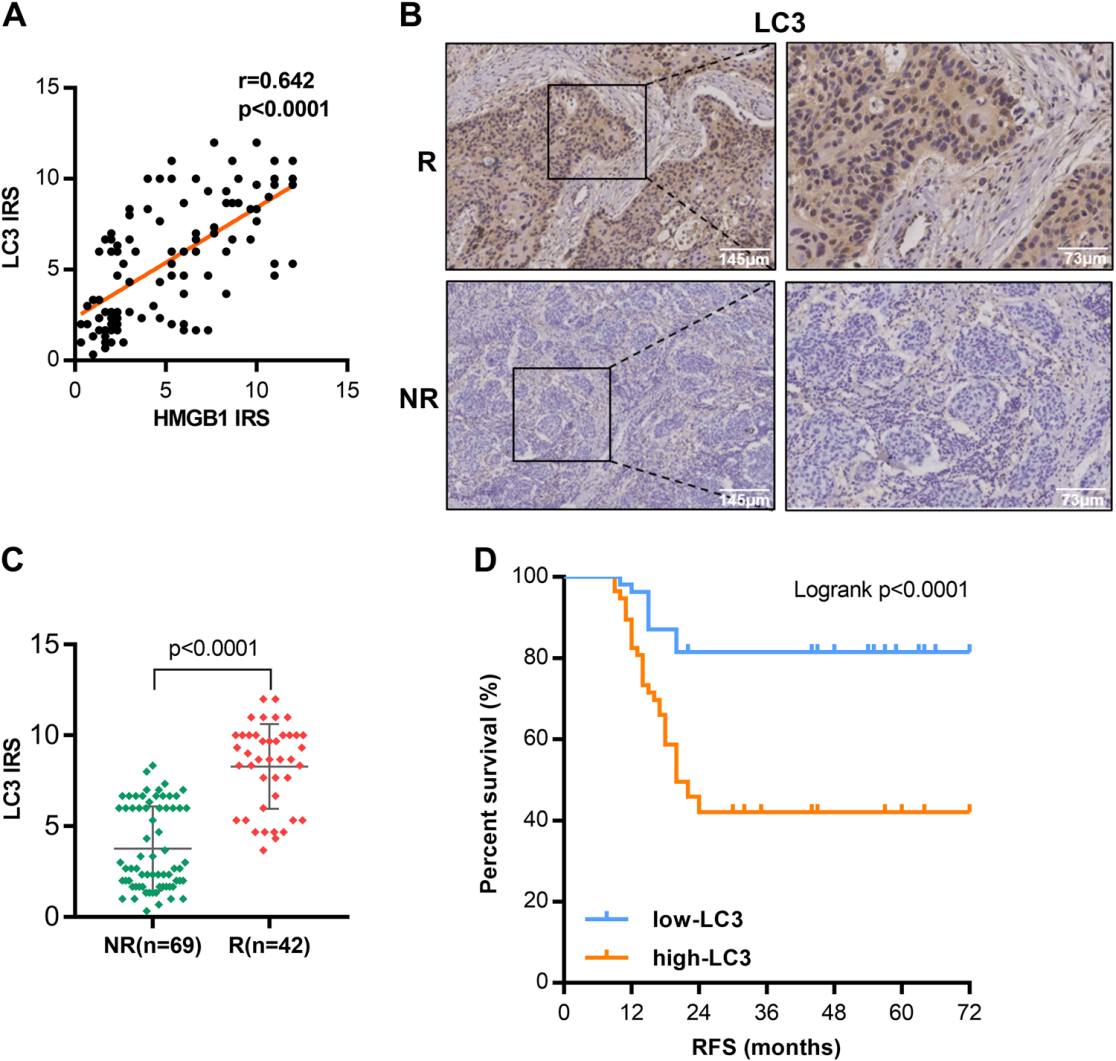
High LC3 expression associates with HMGB1 and poor outcome after postoperative radiotherapy in locally advanced resected ESCC. **a** Correlation of HMGB1 and LC3B protein level in clinical ESCC samples. r, Pearson correlation coefficient. **b** Representative images of IHC staining of LC3B protein in clinical ESCC samples from patients with/without recurrence. R recurrence; NR non-recurrence. **c** Statistical analysis of IRS of LC3 protein in clinical ESCC samples, unpaired Mann–Whitney *U* test. **d** Kaplan-Meier analyses of RFS for ESCC patients with high-level or low-level tumor expression of LC3, Log-rank test

Our cell line-based results showed that inhibiting HMGB1 can increase ESCC’s radiosensitivity by downregulating autophagy. We also investigated the relationship of autophagy status with HMGB1 and radiosensitivity in clinical samples. Anti-LC3 antibody for IHC staining was used to indicate the level of autophagy in clinical samples^16^. We observed protein expression of autophagy marker LC3 was positively correlated with HMGB1 in ESCC tumor tissues (*r* = 0.642, *P* < 0.0001) (Fig. 6a). Concordantly, ESCC patients with in-field recurrence after PORT had higher tumor LC3 expression compared with those without recurrence (*P* < 0.0001) (Fig. 6b, c). Log-rank test showed that patients with higher LC3 had a shorter RFS (*P* < 0.0001) (Fig. 6d).

## Discussion

Radiotherapy is the first preferable treatment for ESCC patients after surgical resection, especially for those in locally advanced stage. However, many ESCC patients were resistant to radiotherapy. Identifying the radioresistance related biomarker help to tailor the treatment. Clarifying the molecular mechanism also helped to develop effective targeted therapies for ESCC patients. In this study we identified the critical role of HMGB1 in mediating response to radiotherapy in ESCC. We proved that HMGB1 in ESCC led to radioresistance through inducing cytoprotective autophagy.

Previous study reported correlation between HMGB1 expression with the stage and the survival of esophageal carcinoma^11,12^. The relationship between HMGB1 expression and survival was also observed in our ESCC cohort. We proved that HMGB1 is associated with recurrence of resected ESCC after PORT. Our results are in good agreement with previous studies in the lower rectal cancer^17^ and epithelial ovarian cancer^18^, that patients with high-HMGB1 expression were more resistance to chemo and radiotherapy. To the best of our knowledge, the correlation of HMGB1 expression and resistance to radiotherapy in ESCC has not been reported previously. HMGB1 level is expected to be one of the potential predictors of radiotherapy response.

We observed elevated autophagy protein expression in clinic samples from radioresistance group, indicating the activation of autophagy in radioresistance ESCC. Responsive autophagy is a well-known mechanism that can protect cells from IR-induced cellular damage^19^. Inhibition of autophagy can increase radiosensitivity of several types of cancer including esophageal carcinoma^20^. In addition, Tang’s study clarified that intracellular HMGB1 activates autophagy by binding to BECN1 and initiating the formation of autophagosomes^13^. It is therefore expected that elevated HMGB1 in ESCC may protect cells from IR damage by enhancing autophagy. Indeed, we observed reduced levels of autophagy in HMGB1-knockdown ESCC cells, accompanied by elevated radiosensitivity. We further proved that the elevated radiosensitivity could be recovered by inducing autophagy. Correlation between HMGB1 and autophagy, as well as their association with recurrence and prognosis were also proved in ESCC clinical samples. Taken together, our results suggested that high-expression of HMGB1 is expected to protect the ESCC’s resistant to radiotherapy possibly through upregulating the autophagy.

Several issues of the current study should be discussed. First, the analysis was conducted only in male group due to low prevalence of ESCC in females and lack of enough female patients for drawing robust conclusion. With the project going on, we foresee the increase of our sample size and expect to expand the analysis to female group. Second, HMGB1 is known to play compartmental role in different cellular locations and it regulation upon autophagy was mainly reported in cytosol. It has been shown that activation of autophagy promotes radioresistance in multiple type of cancer cells^21^, while the role of nuclear HMGB1 in cellular response to irradiation has been little studied. Our results supported that HMGB1 at least partly through activating autophagy in cytosol. It will be interesting to also explore the role of nuclear HMGB1 in the future studies.

In conclusion, our study has shown that HMGB1 expression correlates with recurrence in locally advanced ESCC after esophagectomy and PORT. HMGB1 knockdown increased the radiosensitivity of ESCC both in vitro and in vivo through decreased autophagy. Our investigation supported that high level of HMGB1 expression in tumors may serve as indicators for patients who may benefit little from the PORT. HMGB1 inhibitors may be developed as a combination therapy to help these patients improve the efficacy of radiotherapy.

## Materials and methods

### Patients and clinical specimens

Patients (*n* = 120) with primary locally advanced but resectable ESCC between 2010 and 2011 at the 1st or 2nd Affiliated Hospital of Xi’an Jiaotong University were recruited. Patients were diagnosed according to the International Classification of Disease for Oncology 3rd Edition (ICD-O-3). All patients had undergone radical esophagectomy with lymphadenectomy (R0 resection, R0 = no cancer at resection margins) following with radiotherapy for 5 weeks (D_T_: 50 Gy/25 fractions, 2 Gy/fraction, and 5 fractions/week). No patients had received preoperative chemo/radiotherapy. ESCC patients combined with other tumors, anastomotic leakage or systemic infection were excluded. After surgery, staging of the disease was performed by independent pathologists based on AJCC 8th^22^. Patients at pT3N0M0 or pT1-3N + M0 stage were selected for research. To avoid the potential confounding from gender, only the male patients were included for further research. The formalin-fixed and paraffin-embedded (FFPE) specimens from the cohort were used for analyzed. The Ethics Committee of the Health Science Center of Xi’an Jiaotong University approved the study. Before the study, all patients were informed and signed informed consent. The study was conducted in accordance with the provisions of the Helsinki Declaration of 1975.

RFS was evaluated from the date of esophagectomy to the date of in-field recurrence. In-field recurrence was defined as tumor recurrence within the 95% isodose line and was monitored by imaging examination systems and biopsy.

### Cells lines and culture

ESCC cell lines TE-1 and Eca-109 were obtained directly from Cell Resource Center of Shanghai Institute of Life Sciences, Chinese Academy of Sciences (Shanghai, China) and were not cultured for more than 2 months after receipt. Cells were cultured with RPMI-1640 medium (Thermo Fisher Scientific, Waltham, MA, USA) with 10% fetal bovine serum (FBS) in the 37°C and 5% CO_2_ cell incubator. For starvation assays, cells were washed with phosphate buffered saline (PBS) and then treated with EBSS (Thermo Fisher Scientific) for 4 h. Considering the impact of time-relevant difference of EBSS effect, we did the preliminary time series experiment and determined that 4 h was appropriate to induce autophagy without inhibition of cell viability (Supplementary Fig. S5).

### Subcutaneous xenograft mouse model

All mouse experiments were conducted according to the Public Health Service Policy on Humane Care and Use of Laboratory Animals. Mouse were obtained from and housed in the Medical Experimental Animal Center (Xi’an Jiaotong University, China).

To establish the ESCC xenograft model, TE-1 cells (1 × 10^7^) were injected subcutaneously into the back of each BALB/c nude mouse (male, 4-week old). To explore the role of HMGB1 in radiosensitivity in vivo, three groups were designed: (1) Control shRNA, (2) HMGB1 shRNA, (3) IR plus control shRNA and (4) IR plus HMGB1 shRNA. The IR groups received 2 Gy X-ray IR by linear accelerator (Elekta Instruments, Inc., Stockholm, Sweden) for five consecutive days. The IR treatment was started nine days after transplantation. The length (a) and width (b) of the tumor were measured and recorded every three days and the volume was calculated using the formula V = ab^2^/2. After 15 days, mice were sacrificed and tumors were stripped and weighed. The procedures of the study were approved by the Ethics Committee of the Health Science Center of Xi’an Jiaotong University.

### Western blot

Western blot experiments were performed as previously described^23^. Briefly, protein was extracted from cells, electrophoresed in 15% SDS gel, and then transferred to PVDF membranes. After blocked, the membranes were incubated with primary antibodies (anti-HMGB1, 1:1000, Abcam, Cambridge, UK; anti-LC3, 1:1000, Abcam) overnight and secondary antibodies for 2 h. The signal was detected by ECL Kit (Millipore, MA, USA) and the final scanned images were analyzed using NIH-Image J.

### Clonogenic survival assay to test cells’ radiosensitivity

Clonogenic survival assay were performed as previously described^24^. Cells were incubated into six-well plates and were irradiated at a dose of 0-8 Gy. 14 days after IR, cells were fixed and stained, and clones were counted. Cell survival fraction (SF) was calculated and substituted into the single-hit multitarget model: SF = 1–(1–*e*^ [−*kD*])^*N*. Cell survival curves were then generated using GraphPad Prism 7.0 and radiobiological parameters (*D*_*0*_, *D*_*q*_, *N*, and SF2) were calculated.

### IHC staining analysis of clinical ESCC specimens

FFPE specimens were sectioned 4.5 μm thick and placed in xylene for dewaxing. Specimens were then hydrated by gradient ethanol gradient and placed in citrate buffer for antigen retrieval. After blocked, primary antibodies (anti-HMGB1, 1: 1000, Abcam; anti-LC3, 1:500, Abcam) were incubated overnight. Secondary antibodies were incubated for 1 h and then horseradish peroxidase was added dropwise to perform DAB staining. After soaking in 50% hematoxylin for 6 min, specimens were dehydrated by gradient ethanol, transparented by xylene and mounted with coverslips using mounting medium.

IHC staining was assessed using a light microscope by two independent pathologists on each tissue section according to the IRS established by Remmele and Stegner^25^. ESCC specimens staining was assessed by using staining of the normal esophageal epithelial cells as internal control. IRS combines a score for staining intensity from 0–3 (0, no color reaction; 1, mild; 2, moderate; 3, intense) multiplied with the score for the percentage of positive cells from 0–4 (0, no positive cells; 1, <10%; 2, 10–50%; 3, 51–80%; 4, >80%). The level of protein expression was identified as low-expression (IRS < 6) and high-expression (IRS ≥ 6) based on the IRS.

### siRNA transfection

HMGB1 siRNA (siHMGB1 group) and scrambled siRNA (control group) were purchased from Gene Pharma (Shanghai, China). Cells were transfected with siRNA using Micropoly-transfecter^TM^ Tissue Reagent (Micropoly, Nantong, China) according to the manufacturers’ instruction. Six hour later, cells were washed with PBS and cultured with complete medium in the incubator for 48 h.

### shRNA adenovirus infection

HMGB1 shRNA (shHMGB1 group) and scrambled shRNA (control group) were purchased from Hanheng Biotechnology (Hanheng Biotechnology Co., Ltd., Shanghai, China). Cells were plated into six-well plates and infected with shRNA adenovirus according to the manufacturer’s instructions. Six hour later, cells were washed with PBS and cultured with complete medium in the incubator for 48 h.

### Transmission electron microscopic inspection

Cells were fixed with the Electron Microscope Fixative (Servicebio Technology CO., LTD, Wuhan, China) for 2 h. The fixed samples were dehydrated with a series of acetone, embedded and solidified. Ultrathin sections (50 nm) were prepared and carefully placed on the support membrane. The intracellular structures were observed using TEM HT7700 (Hitachi, Tokyo, Japan).

### Autophagy flux monitor

The mRFP-GFP-LC3 adenovirus vectors were used to monitor autophagy flux (Hanheng Biotechnology Co., Ltd., Shanghai, China). LC3 is tracked by red mRFP and green GFP, while GFP is sensitive to acidity. When lysosomes and autophagosomes were fused into autolysosomes, the signal of GFP fluorescence quenching and mRFP fluorescence remained unchanged. Therefore, the red signal indicates autolysosomes and the yellow signal indicates autophagosomes.

Cells were seeded in a 35-mm laser confocal culture dish and mRFP-GFP-LC3 adenovirus was infected according to the manufacturer’s instructions. 6 h later, cells were washed with PBS and cultured with complete medium in the incubator for 48 h. Laser confocal fluorescence microscopy IX83 (Olympus, Tokyo, Japan) was used to observe the autophagy flux and LC3 puncta.

### Statistical analysis

Measurement data between two groups were compared by Mann–Whitney *U* test or *t* test where appropriate. The correlation between HMGB1 expression and recurrence rate was performed using the Chi-square test. Log-rank test was used to determine the significance of Kaplan-Meier curves. To eliminate the confounding effect, univariable Cox proportional hazard model and logistic regression were used to filter for associated factors of RFS. Then multivariable Cox proportional hazard model and logistic regression were used to analyze association between HMGB1 expression and RFS adjusting for age, tumor location, histology grade and pTN stage. Statistical analysis software SPSS 20.0 (SPSS, Inc., IL, USA) and GraphPad Prism 7.0 (GraphPad Software, Inc., CA, USA) were used. All data were presented as mean ± SEM of three or more experiments. *P* *<* 0.05 was considered statistically significant.

## Supplementary information

Supplementary Table S1

Supplementary Table S2

Supplementary Table S3

Supplementary Figure S1

Supplementary Figure S2

Supplementary Figure S3

Supplementary Figure S4

Supplementary Figure S5

Supplemental figure legends

## References

[1] Fitzmaurice C (2015). The global burden of cancer 2013. JAMA Oncol..

[2] Liang H, Fan JH, Qiao YL (2017). Epidemiology, etiology, and prevention of esophageal squamous cell carcinoma in China. Cancer Biol. Med..

[3] Pennathur A, Gibson MK, Jobe BA, Luketich JD (2013). Oesophageal carcinoma. Lancet.

[4] Teniere P, Hay JM, Fingerhut A, Fagniez PL (1991). Postoperative radiation therapy does not increase survival after curative resection for squamous cell carcinoma of the middle and lower esophagus as shown by a multicenter controlled trial. French University Association for Surgical Research. Surg., Gynecol. Obstet..

[5] Fok M, Sham JS, Choy D, Cheng SW, Wong J (1993). Postoperative radiotherapy for carcinoma of the esophagus: a prospective, randomized controlled study. Surgery.

[6] Chen J (2010). Postoperative radiotherapy improved survival of poor prognostic squamous cell carcinoma esophagus. Ann. Thorac. Surg..

[7] Xiao ZF (2003). Value of radiotherapy after radical surgery for esophageal carcinoma: a report of 495 patients. Ann. Thorac. Surg..

[8] Lotze MT, Tracey KJ (2005). High-mobility group box 1 protein (HMGB1): nuclear weapon in the immune arsenal. Nat. Rev. Immunol..

[9] Shrivastava S (2016). The role of HMGB1 in radioresistance of bladder cancer. Mol. Cancer Ther..

[10] Ke S (2015). Downregulation of high mobility group box 1 modulates telomere homeostasis and increases the radiosensitivity of human breast cancer cells. Int J. Oncol..

[11] Chuangui C, Peng T, Zhentao Y (2012). The expression of high mobility group box 1 is associated with lymph node metastasis and poor prognosis in esophageal squamous cell carcinoma. Pathol. Oncol. Res..

[12] Chen C, Cui L, Tang P, Yu Z (2013). Clinical significance of serum high-mobility group box 1 detection in esophageal squamous cell carcinoma. Zhonghua Wei Chang Wai Ke Za Zhi.

[13] Tang D (2010). Endogenous HMGB1 regulates autophagy. J. Cell Biol..

[14] Chen X (2017). Autophagy enhanced the radioresistance of non-small cell lung cancer by regulating ROS level under hypoxia condition. Int J. Radiat. Biol..

[15] Chaachouay H (2011). Autophagy contributes to resistance of tumor cells to ionizing radiation. Radiother. Oncol..

[16] Klionsky DJ (2016). Guidelines for the use and interpretation of assays for monitoring autophagy (3rd edition. Autophagy.

[17] Hongo K (2015). Immunohistochemical detection of high-mobility group box 1 correlates with resistance of preoperative chemoradiotherapy for lower rectal cancer: a retrospective study. World J. Surg. Oncol..

[18] Machado LR (2017). High mobility group protein B1 is a predictor of poor survival in ovarian cancer. Oncotarget.

[19] Wang, F., et al. SMAD4 gene mutation renders pancreatic cancer resistance to radiotherapy through promotion of autophagy. *Clin. Cancer Res.* **24**, 3176–3185 (2018). 10.1158/1078-0432.CCR-17-3435 PMC6345154 29602802

[20] Tao, H., et al. Autophagy inhibition enhances radiosensitivity of Eca109 cells via the mitochondrial apoptosis pathway. *Int. J. Oncol.* **52**, 1853–1862 (2018). 10.3892/ijo.2018.4349 PMC5919709 29620258

[21] Sharma K, Goehe R, Beckta JM, Valerie K, Gewirtz DA (2014). Autophagy and radiosensitization in cancer. EXCLI J..

[22] Rice TW, Ishwaran H, Ferguson MK, Blackstone EH, Goldstraw P (2017). Cancer of the esophagus and esophagogastric junction: an eighth edition staging primer. J. Thorac. Oncol..

[23] Gong T (2017). PTENP1 inhibits the growth of esophageal squamous cell carcinoma by regulating SOCS6 expression and correlates with disease prognosis. Mol. Carcinog..

[24] Huang S (2013). Inhibition of microRNA-21 increases radiosensitivity of esophageal cancer cells through phosphatase and tensin homolog deleted on chromosome 10 activation. Dis. Esophagus..

[25] Remmele W, Stegner HE (1987). [Recommendation for uniform definition of an immunoreactive score (IRS) for immunohistochemical estrogen receptor detection (ER-ICA) in breast cancer tissue]. Pathologe.

