# Supplementary material for: High mobility group box 1 promotes radioresistance in esophageal squamous cell carcinoma cell lines by modulating autophagy
Source: Cell Death Dis. 2019 Feb 12;10(2):136. doi: 10.1038/s41419-019-1355-1 (PMC6372718; doi:10.1038/s41419-019-1355-1)
Supplement: Supplementary file 1 — Supplementary Table S1 [file 41419_2019_1355_MOESM1_ESM.docx]

**Supplementary Table S1 Patients and Treatment Characteristics**

| **Characteristic** | **No. (%) of Patients** |
| --- | --- |
| **Age(median)** | 60y (40-73y) |
| **Sex** |  |
| Male | 111(100%) |
| **Histology** |  |
| SC | 111(100%) |
| **Tumor location** |  |
| Upper Thoracic | 35(31.5%) |
| Middle Thoracic | 58(52.3%) |
| Lower Thoracic | 18(16.2%) |
| **Grade** |  |
| G1 | 57(51.4%) |
| G2-3 | 54(48.6%) |
| **pTN stage** |  |
| pT3N0 | 38(34.2%) |
| pT1-3N+ | 73(65.8%) |
| **Type of surgery** |  |
| radical esophagectomy | 111(100%) |
| **Margin status** |  |
| R0 | 111(100%) |
| **PORT dose** |  |
| 50 Gy | 111(100%) |

Abbreviation: SC, squamous cell carcinoma; PORT, postoperative radiotherapy.
