# Supplementary material for: High mobility group box 1 promotes radioresistance in esophageal squamous cell carcinoma cell lines by modulating autophagy
Source: Cell Death Dis. 2019 Feb 12;10(2):136. doi: 10.1038/s41419-019-1355-1 (PMC6372718; doi:10.1038/s41419-019-1355-1)
Supplement: Supplementary file 2 — Supplementary Table S2 [file 41419_2019_1355_MOESM2_ESM.docx]

**Supplementary Table S2 Multivariate analyses of variables associated with RFS**

| **Characteristic** |  | | **RFS** | | |
| --- | --- | --- | --- | --- | --- |
|  |  | ***P*** | | **HR*** | **95% CI** |
| **Age** |  |  | |  |  |
| <60y |  | Reference | | 1 |  |
| ≥60y |  | 0.674 | | 0.873 | 0.463-1.645 |
| **Tumor location** |  |  | |  |  |
| Upper Thoracic |  | Reference | | 1 |  |
| Middle Thoracic |  | 0.733 | | 0.885 | 0.440-1.782 |
| Lower Thoracic |  | 0.565 | | 0.732 | 0.253-2.117 |
| **Histology Grade** |  |  | |  |  |
| G1-2 |  | Reference | | 1 |  |
| G3 |  | <0.001 | | 8.042 | 3.417-18.930 |
| **pTN stage** |  |  | |  |  |
| pT3N0 |  | Reference | | 1 |  |
| pT1-3N+ |  | <0.001 | | 7.843 | 3.331-18.468 |
| **HMGB1** |  |  | |  |  |
| Low |  | Reference | | 1 |  |
| High |  | <0.001 | | 3.832 | 1.876-7.826 |

Abbreviation: RFS, relapse-free survival; HR, hazard ratio; CI, confidence interval.

*****HRs were derived from multivariable Cox proportional hazards regression analysis, and models were adjusted for all confounding factors listed in the table.
