# Supplementary material for: High mobility group box 1 promotes radioresistance in esophageal squamous cell carcinoma cell lines by modulating autophagy
Source: Cell Death Dis. 2019 Feb 12;10(2):136. doi: 10.1038/s41419-019-1355-1 (PMC6372718; doi:10.1038/s41419-019-1355-1)
Supplement: Supplementary file 3 — Supplementary Table S3 [file 41419_2019_1355_MOESM3_ESM.docx]

**Supplementary Table S3 Association between tumor HMGB1 expression and** **patients’ survival**

| **Model** | **RFS** | | |  | **OS** | | |
| --- | --- | --- | --- | --- | --- | --- | --- |
|  | **OR*** | **95% CI** | ***P*** |  | **OR*** | **95% CI** | ***P*** |
| **Unadjusted** | 2.091 | 1.618-2.701 | <0.001 |  | 1.604 | 1.278-2.012 | <0.001 |
| **Adjusted** | 2.310 | 1.582-3.373 | <0.001 |  | 1.553 | 1.191-2.024 | <0.001 |

Abbreviation: RFS, relapse-free survival; OS, overall survival; OR, odds ratio; CI, confidence interval.

*HRs were derived from logistic regression analysis, and models were adjusted for all confounding factors including age, tumor location, histology grade and pTN stage.
