# Supplementary figures and images for: High mobility group box 1 promotes radioresistance in esophageal squamous cell carcinoma cell lines by modulating autophagy
Source: Cell Death Dis. 2019 Feb 12;10(2):136. doi: 10.1038/s41419-019-1355-1 (PMC6372718; doi:10.1038/s41419-019-1355-1)

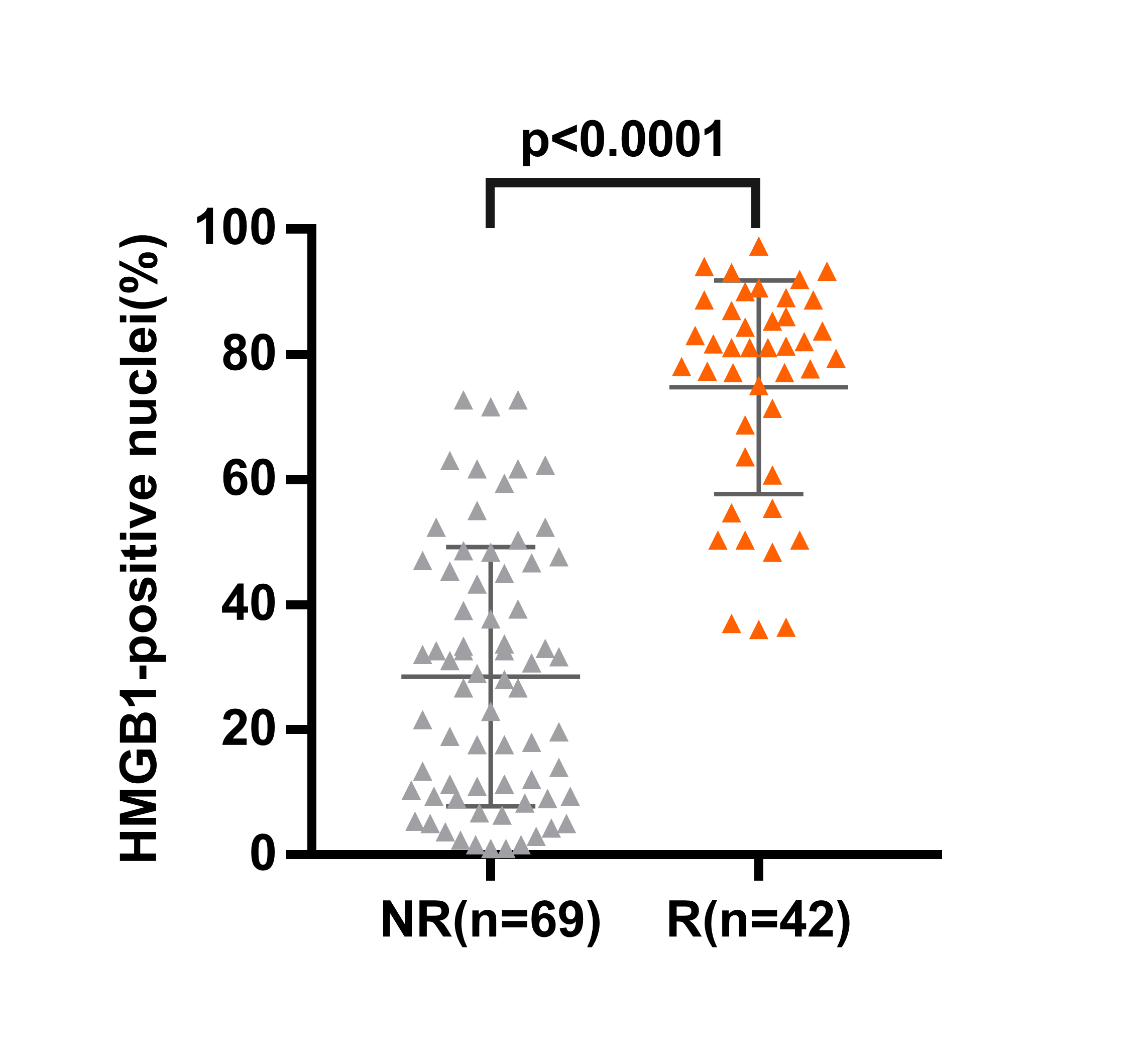

Supplement: Supplementary file 4 — Supplementary Figure S1 [file 41419_2019_1355_MOESM4_ESM.tif]

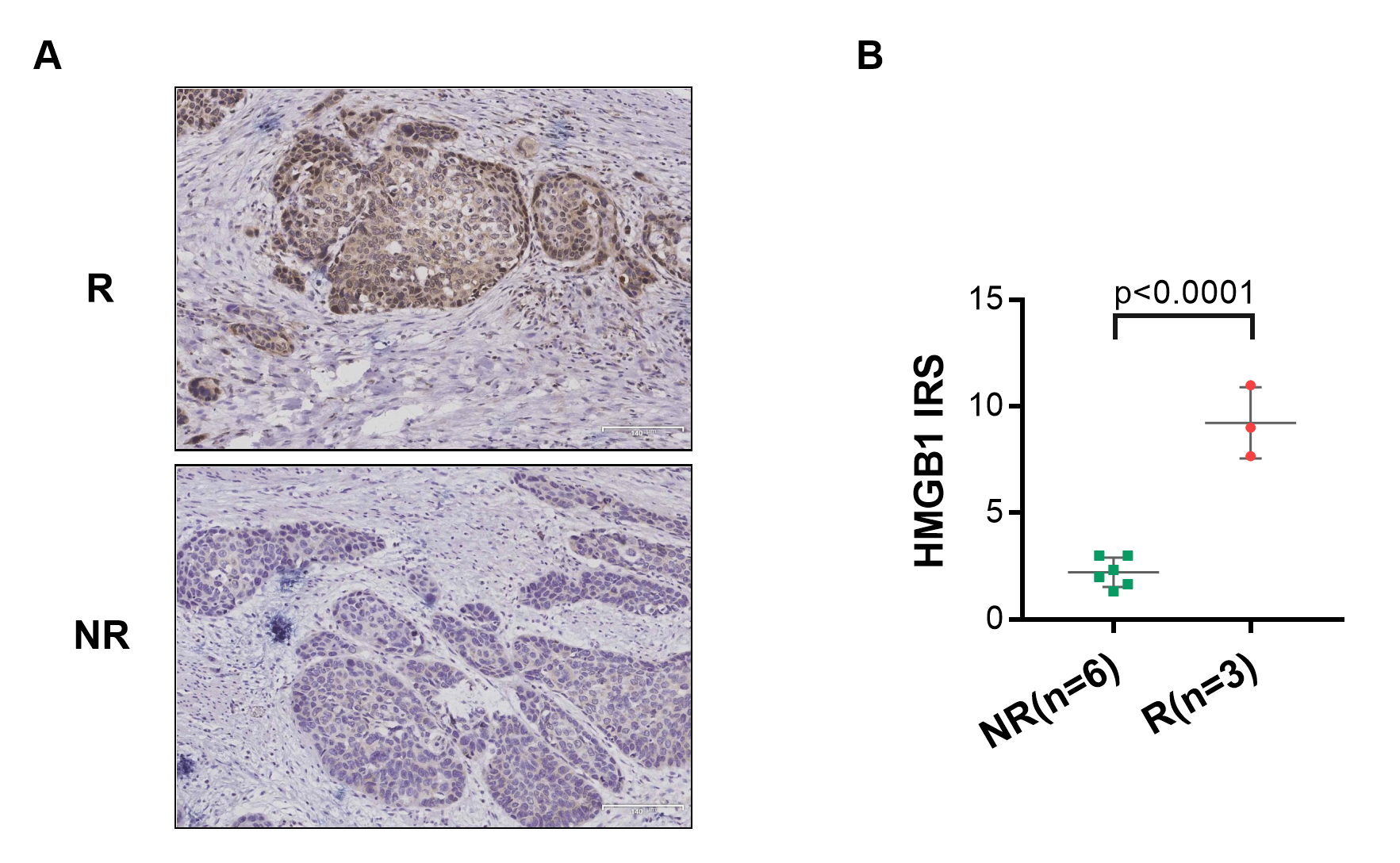

Supplement: Supplementary file 5 — Supplementary Figure S2 [file 41419_2019_1355_MOESM5_ESM.tif]

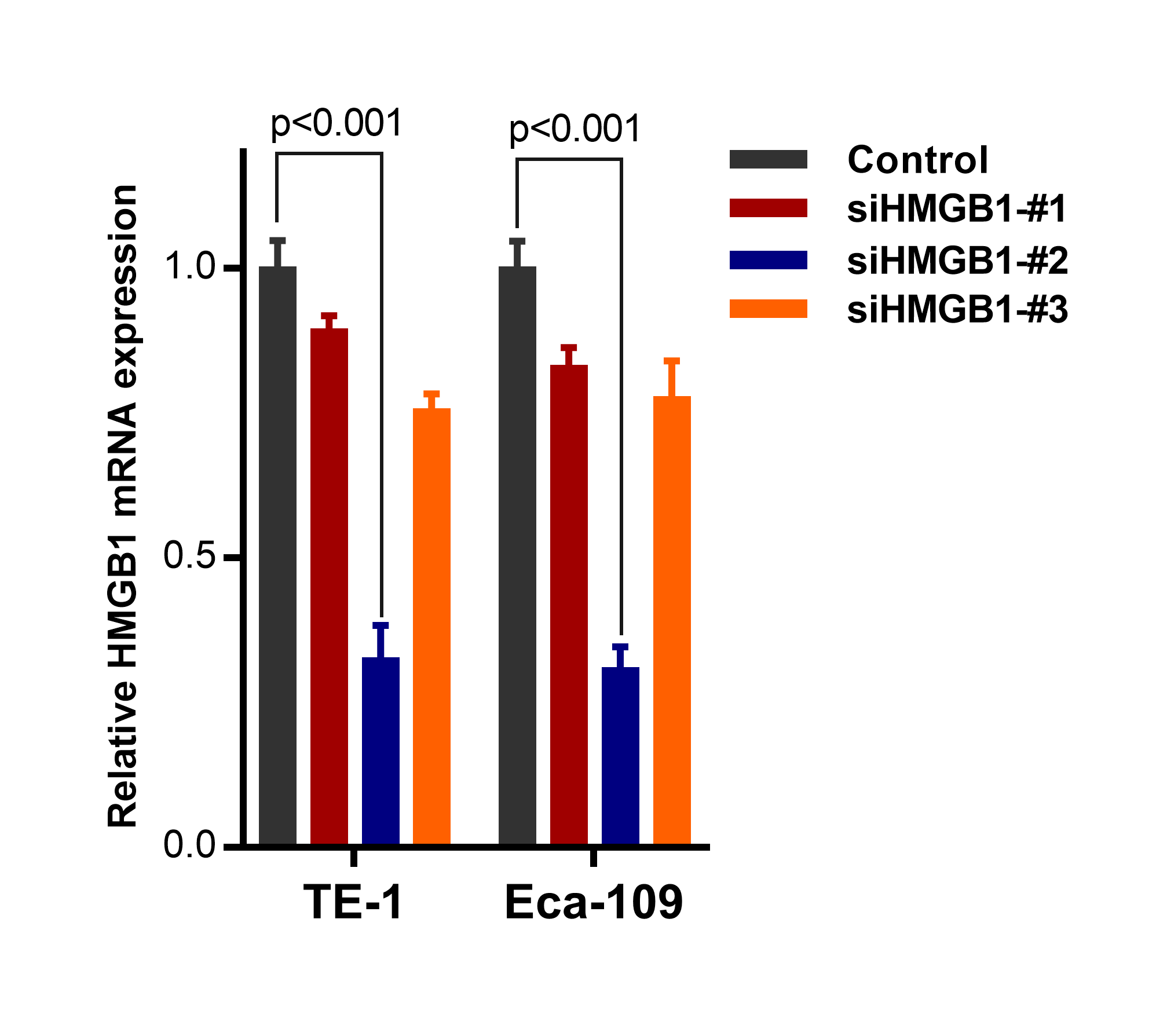

Supplement: Supplementary file 6 — Supplementary Figure S3 [file 41419_2019_1355_MOESM6_ESM.tif]

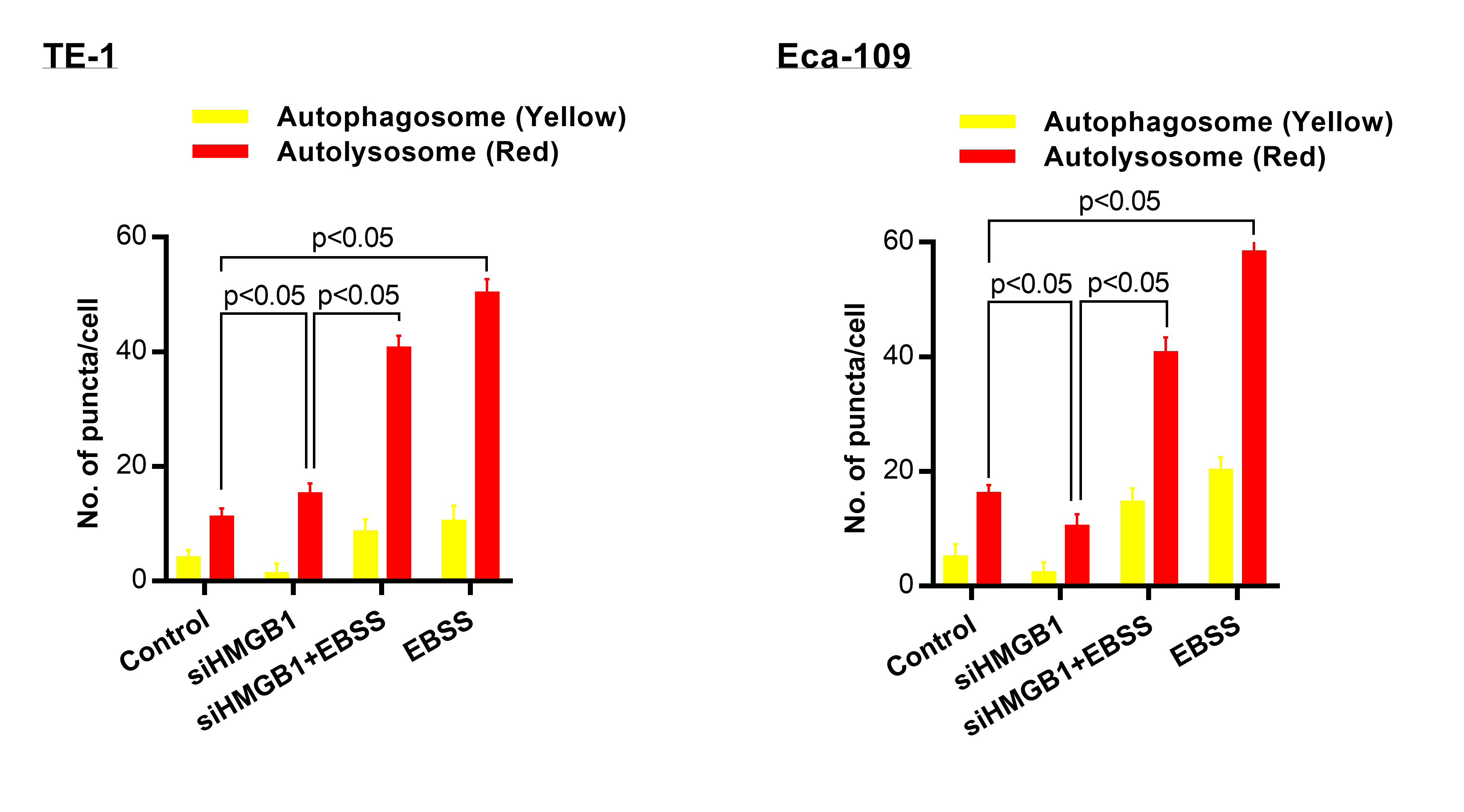

Supplement: Supplementary file 7 — Supplementary Figure S4 [file 41419_2019_1355_MOESM7_ESM.tif]

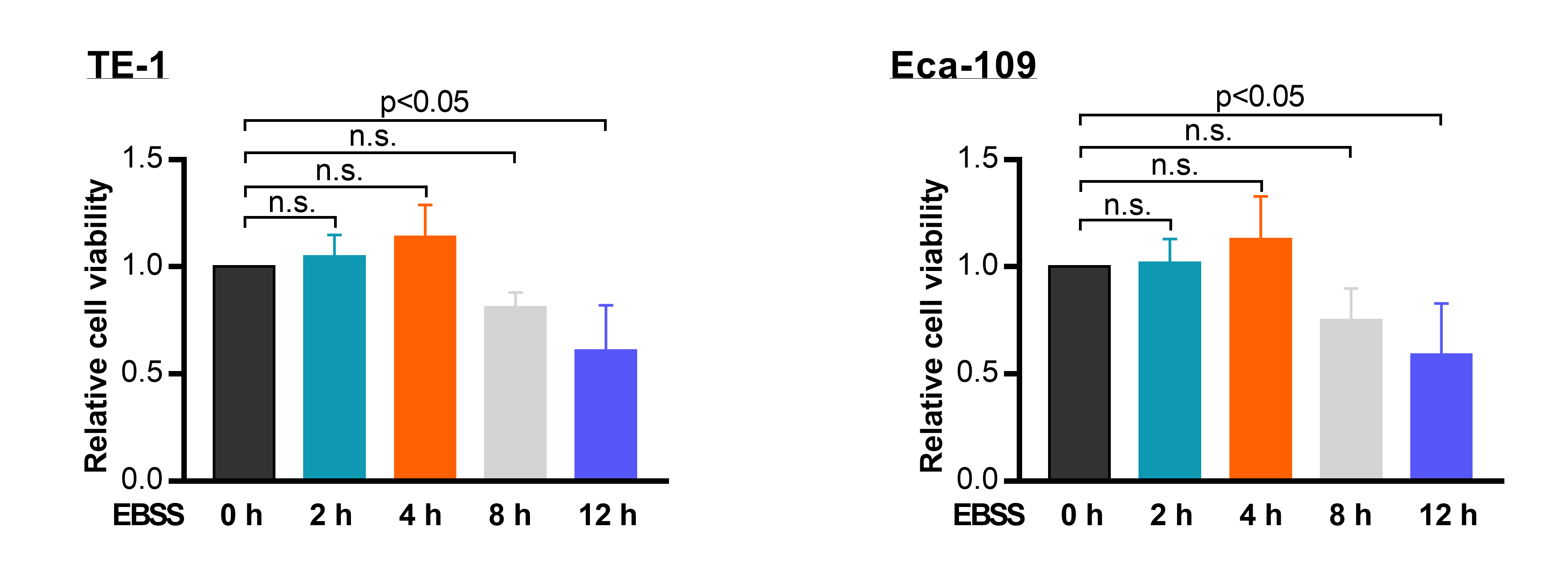

Supplement: Supplementary file 8 — Supplementary Figure S5 [file 41419_2019_1355_MOESM8_ESM.tif]
