# Supplementary material for: High mobility group box 1 promotes radioresistance in esophageal squamous cell carcinoma cell lines by modulating autophagy
Source: Cell Death Dis. 2019 Feb 12;10(2):136. doi: 10.1038/s41419-019-1355-1 (PMC6372718; doi:10.1038/s41419-019-1355-1)
Supplement: Supplementary file 9 — Supplemental figure legends [file 41419_2019_1355_MOESM9_ESM.docx]

**Supplementary Figure Legends**

**Fig. S1** HMGB1-positive nuclei (%) in male ESCC samples (n=111), unpaired Mann-Whitney U test. R, recurrence; NR, non-recurrence.

**Fig. S2** HMGB1 expression in female ESCC samples (n=9). **(A)** Representative image of immunohistochemistry of HMGB1 protein. **(B)** Immunoreactivity score (IRS) of HMGB1 protein, unpaired Mann-Whitney U test. R, recurrence; NR, non-recurrence.

**Fig. S3** Knockdown efficiency of different HMGB1 siRNAs. Real-time PCR was performed to detect expression of HMGB1 mRNA in siHMGB1-transfected cells.

**Fig. S4** Quantification for autophagosomes and autophagosomes by laser confocal fluorescence microscopy analysis. ESCC cells stably expressing mRFP-GFP-LC3 protein were transfected with siHMGB1 and/or treated with starvation (EBSS for 4 hours).

**Fig. S5** Cell viability of TE-1 cells and Eca-109 cells co-cultured with EBSS. n.s, not significant.
